# Perforated viscus as first presentation of Crohn’s disease: a case report

**DOI:** 10.1093/jscr/rjab415

**Published:** 2021-09-29

**Authors:** Jia Hui Lee, Tze Tong Tey, Fung Joon Foo, Frederick Koh

**Affiliations:** Department of General Surgery, Sengkang General Hospital, Singapore, Singapore; Department of Gastroenterology, Sengkang General Hospital, Singapore, Singapore; Department of General Surgery, Sengkang General Hospital, Singapore, Singapore; Department of General Surgery, Sengkang General Hospital, Singapore, Singapore

## Abstract

Bowel perforation as the first presentation of inflammatory bowel disease is rare and unusual in young patients. A previously asymptomatic 21-year-old Asian male presented with perforated small bowel secondary to previously undiagnosed Crohn’s disease. He underwent an exploratory laparotomy and subsequent small bowel resection and was commenced on mesalazine post-operation. He recovered well with subsequent regular follow-up with gastroenterology. The main management of Crohn’s disease is multidisciplinary in nature, and collaboration between different disciplines is inherent with the aim of reducing symptoms and maximizing patient quality of life.

## INTRODUCTION

The perforation of bowel as the first presentation of inflammatory bowel disease is a rare occurrence reported in about 0.15–3% of the literature [1, 2] and is especially unusual in a young patient less than 30 years of age [2]. We present a case of a previously asymptomatic 21-year-old Asian male who presented with perforated small bowel secondary to previously undiagnosed Crohn’s disease, with a review of the literature.

## CASE REPORT

A 21-year-old Singaporean Chinese male was admitted for sudden onset generalized abdominal pain and one episode of non-bilious, non-bloody vomiting. He reported no history of fish bone ingestion, no diarrhoea and no loss of weight. He denied any history of joint pains, eye or dermatological symptoms. There was no family history of gastrointestinal disorders.

On presentation, his heart rate was 103, and he had a temperature of 37.7, with other vital signs within normal limits. On examination, he possessed a guarded abdomen, with generalized tenderness on palpation. His white cell count was 18.6 × 10^9^ (reference range, 3.8–10.0 × 10^9^/l) on presentation.

**Figure 1:**
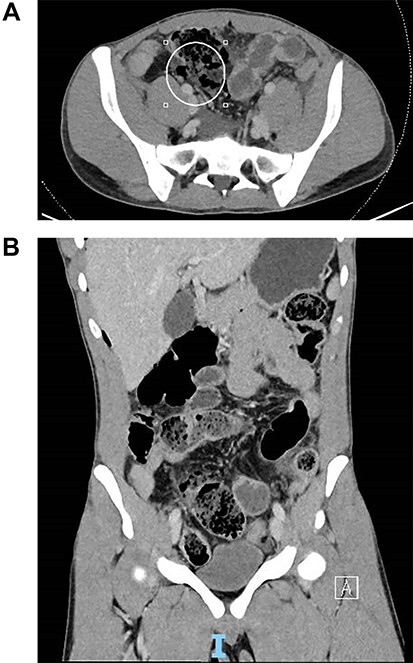
(**A**) Coronal slice of computed tomography (CT) image of small bowel perforation at distal ileum. (**B**) Sagittal slice of CT image of small bowel perforation at distal ileum.

A chest x-ray noted a small amount of subdiaphragmatic air. A computed tomography showed small bowel perforation at the distal ileum with scattered segmental wall thickening and mild surrounding fat stranding involving the transverse colon, terminal ileum and distal ileum as shown in Fig. 1a and b.

The patient was commenced on intravenous ceftriaxone and metronidazole and underwent an exploratory laparotomy, small bowel resection and primary anastomosis.

**Figure 2:**
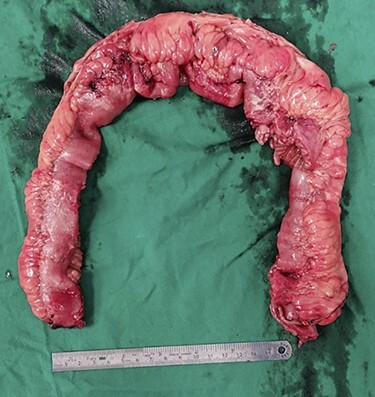
Uncut specimen of small bowel showing fat creeping.

**Figure 3:**
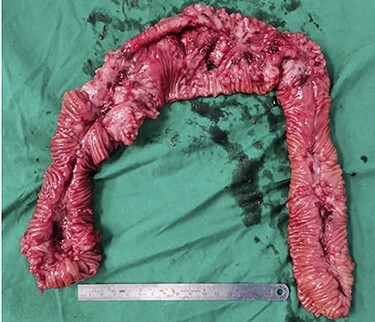
Cut specimen of small bowel showing 2 cm defect into the mesentery and another more proximal with 1 cm defect at the anti-mesentery border 3 cm away from the above 30 cm from IC valve.

Intraoperatively, fat creeping of the small bowel was noted as seen in Fig. 2. There were two small bowel perforations noted, first with 2 cm defect into the mesentery and another more proximal with 1 cm defect at the anti-mesentery border 3 cm away from the above 30 cm from ileocaecal (IC) valve as depicted in Fig. 3. There was unhealthy and indurated bowel and mesentery from 15 cm from IC valve to 70 cm from IC valve (total length resected 55 cm). There were also skip areas of ischaemic enteritis especially surrounding the area of perforation.

Histology of the specimen showed perforation into the mesentery and into peritoneal space, with granulomatous inflammation suggestive of Crohn’s disease. The acid-fast bacilli test for tuberculosis returned negative. The patient recovered uneventfully, and in view of the diagnosis of Crohn’s disease, was commenced on mesalazine 1 g twice daily on discharge.

## DISCUSSION

In this case, it is quite notable that the patient reported no symptoms until his first presentation to the hospital with perforated bowel. Free perforation of the bowel as first presentation of Crohn’s disease is rare with an incidence of 3.4%, with 86.1% of perforations involving the ileum [2]. It is accepted that Western and Eastern phenotypes do differ, with patients from Eastern populations leaning towards a male predominance, ileocolonic disease, and a lower association with cigarette smoking, familial aggregation and extraintestinal disease compared to Western populations [3]. A higher incidence of free perforation has also been described in both Korean and Japanese populations at about 6.5–6.8% compared to the 1–3% described in the Western population. Due to the prevalence of patients over 30 years of age who presented with perforation, the theory is that this higher incidence in the Korean and Japanese population may be linked to diagnostic and treatment delays [2].

There are other diagnostic considerations for Asian countries. Intestinal tuberculosis can mimic the clinical manifestations of inflammatory bowel disease which can result in a misdiagnosis rate of 50–70%, and such a misdiagnosis can lead to complications once immunosuppressant medications are commenced to treat inflammatory bowel disease, as this may lead to reactivation or disseminated tuberculosis [4]. Hence, it is important to exclude tuberculosis before the initiation of biological agents to induce and maintain remission of Crohn’s disease.

When it comes to surgical management, between 70 and 90% of patients will require surgery during their lifetime, with up to 39% requiring repeated surgery. Indications for surgery are complications derived from strictures, intra-abdominal and perianal abscess and fistulae, bowel perforation, bleeding and malignancy. The main aspect of surgical management is to limit the amount of bowel resected as much as possible to avoid complications like short bowel syndrome [5]. Young patients of less than 25 years in age are also at risk of depression, anxiety and isolation due to the chronicity, relapsing nature and intrusiveness of their symptoms [6]. The main management of Crohn’s disease is multidisciplinary in nature, and collaboration between different disciplines is inherent with the aim of reducing symptoms and maximizing patient quality of life.

## STATEMENTS

All authors are in agreement of the content of this manuscript.

## STATEMENT OF ETHICS

Written informed consent has been obtained from the patient for publication of details of their medical case and accompanying images. Ethics approval was not required.

## AUTHOR CONTRIBUTIONS

All authors discussed the results and contributed to the final manuscript. Dr Tze Tong Tey, Dr Fung Joon Foo and Dr Frederick Koh contributed to the management of the patient. Dr Jia Hui Lee wrote the manuscript. All authors approve the final version of the manuscript.

## CONFLICT OF INTEREST STATEMENT

The authors have no conflict of interest/competing interests to declare. This manuscript has not been published previously and is not under consideration elsewhere.

## FUNDING

This research received no specific grant from any funding agency in the public, commercial or not-for-profit sectors.
